# Knockdown of mitochondrial threonyl-tRNA synthetase 2 inhibits lung adenocarcinoma cell proliferation and induces apoptosis

**DOI:** 10.1080/21655979.2022.2037368

**Published:** 2022-02-19

**Authors:** Hui Tian, Hao Yan, Yong Zhang, Qiaofen Fu, Chunyan Li, Juan He, Hui Li, Yong Zhou, Youguang Huang, Rongqing Li

**Affiliations:** a Department of Radiation Oncology, The First Affiliated Hospital of Kunming Medical University, Kunming, Yunnan, China; b Department of Head and Neck Surgery Section II, The Third Affiliated Hospital of Kunming Medical University (Tumor Hospital of Yunnan Province), Kunming, Yunnan, China; c Department of Dermatology and Venereology, First Affiliated Hospital of Kunming Medical University, Kunming, Yunnan, China; d Division Department of Thoracic Surgery Organization, The First Affiliated Hospital of Kunming Medical University, Kunming, Yunnan, China; e Centre for Experimental Studies and Research, First Affiliated Hospital of Kunming Medical University, Kunming, Yunnan, China; f Department of Yunnan Tumor Research Institute, The Third Affiliated Hospital of Kunming Medical University (Tumor Hospital of Yunnan Province), Kunming, China

## Abstract

Lung cancer is a significant global burden. Aminoacyl-tRNA synthetases (aaRSs) can be reliably identified by the occurrence and improvement of tumors. Threonyl-tRNA synthetase (TARS) and mitochondrial threonyl-tRNA synthetase 2 (TARS2) are both aaRSs. Many studies have shown that TARS are involved in tumor angiogenesis and metastasis. However, TARS2 has not yet been reported in tumors. This study explored the role of TARS2 in the proliferation and apoptosis of lung adenocarcinoma (LUAD). TARS2 expression in lung adenocarcinoma and non-cancerous lung tissues was detected via immunohistochemistry. Cell proliferation was detected using MTS, clone formation, and EdU staining assays. Flow cytometry was used to detect cell cycle, mitochondria reactive oxygen species (mROS) production, and apoptosis. Mitochondrial membrane potential (MMP ΔΨm) was detected using JC-1 fluorescent probes. Cell cycle, apoptosis-related pathway, and mitochondrial DNA (mtDNA) -encoded protein expression was detected via Western blotting. Finally, the effect of TARS2 on tumor growth was examined using a xenotransplanted tumor model in nude mice. We found that TARS2 was highly expressed in lung adenocarcinoma tissues and associated with poor overall survival (OS). Mechanistic analysis showed that knockdown of TARS2 inhibited proliferation through the retinoblastoma protein (RB) pathway and promoted mROS-induced apoptosis. Knockdown of TARS2 inhibits tumor growth in a xenotransplanted tumor model. TARS2 plays an important role in LUAD cell proliferation and apoptosis and may be a new therapeutic target.

## Graphical abstract

**Figure:**
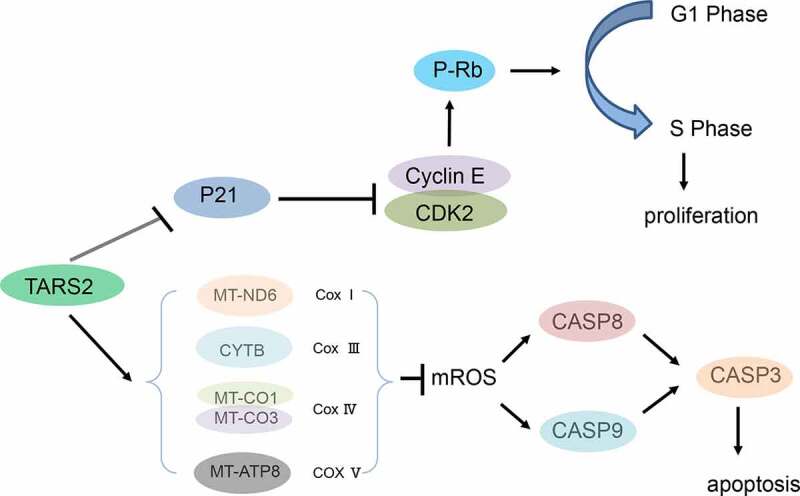

## 1. Introduction

Lung cancer is a huge burden worldwide and is responsible for the most cancer-related deaths; lung adenocarcinoma (LUAD) is the main pathological type [1–5]. Although the mortality rate of LUAD has decreased, the overall survival of patients remains satisfactory [1]. Therefore, clarifying the mechanism of the malignant biological behavior of LUAD and discovering its key signaling targets is of great value.

Tumorigenesis involves abnormal gene expression [6,7]. Many proto-oncogenes maintain normal life processes in the body; however, cancer is often induced if they are abnormally activated [8–11]. The classic function of aminoacyl-tRNA synthetases (aaRSs) is to conjugate amino acids to tRNAs [12]. However, aaRSs are also involved in RNA synthesis, inflammation, apoptosis, and so on [13]. Many studies have linked aaRS function with cancer [14].

TARS and TARS2 are aaRS members that encode cytoplasmic and mitochondrial threonyl-tRNA synthetases, respectively [15]. TARS is abnormally expressed in many types of tumors and is associated with tumor angiogenic markers, metastasis, and poor patient prognosis [16,17]. However, the function of TARS2 in cancer has not been reported. Current research shows that P282L mutation of TARS2 causes mitochondrial encephalomyopathies in humans [18,19]. Mitochondrial DNA (mtDNA) mutations or loss is characteristic of mitochondrial encephalomyopathies [20].

We speculated that TARS2 acts as an oncogene in lung adenocarcinoma, promotes cell proliferation, and reduces apoptosis. Our work aims to reveal the role and possible mechanism of TARS2 in the proliferation and apoptosis of lung adenocarcinoma.

## 2. Methods and methods

### 2.1. Clinical samples

This study included archival formalin-fixed paraffin-embedded specimens from 152 patients (90 women and 62 men) with primary LUAD who received surgical treatment at the Third Affiliated Hospital of Kunming Medical University from 2009 to 2015 and fresh tissue samples from five patients (3 women and 2 men) with primary LUAD who received surgical treatment at the First Affiliated Hospital of Kunming Medical University between 2019 and 2020. Their ages ranged from 28 to 79 years. No patients underwent any other treatment. All patients were free from infectious diseases. The samples were tumor tissue and distally paired normal tissue obtained intraoperatively from patients with LUAD. Written informed consent was obtained from all patients. The Ethics Committee of Kunming Medical University approved the study protocol. The clinical procedures were performed in accordance with the Declaration of Helsinki [21].

### 2.2. Immunohistochemistry (IHC)

Patient and xenograft tumor tissue samples were fixed with formaldehyde (4%) for 24 h. These samples were then embedded in paraffin and cut into 4 μm sections. The 4 μm paraffin tissue sections were toasted at 70°C for 2 h; the xylene was dewaxed, and the gradient alcohol was hydrated. Citrate solution (pH 6.0) was used to repair the antigen. Endogenous peroxidase was blocked with hydrogen peroxide (0.3%), and nonspecific antigens were blocked with sheep serum (2.5%). Sections were incubated with primary antibodies at 28°C for 80 min. The sections were stained with solution A in DAB chromogenic solution, hematoxylin was used to stain the nuclei, and neutral gum was used to seal the sections. The TARS2 protein was stained brown and the nuclei were stained blue. The TARS2 score was defined as the intensity score (no-stain: 0, light-yellow: 1, brown-yellow: 2, brown: 3) multiplied by the percentage area stained (less than 10%: 1, 10–49%: 2, 50–74%: 3, 75–100%: 4). A score of > 4 was considered positive [22].

### 2.3. Cell culture

A549 and PC-9 cells were purchased from Shanghai Cell Bank. A549 cells were cultured in DMEM F12 (with 10% fetal bovine serum), and PC-9 was cultured in RPMI 1640 (with 10% fetal bovine serum) medium. All media were supplemented with 100 μM penicillin and streptomycin [23].

### 2.4. Cell infection

PC-9 and A549 cells were seeded in 6-well plates (20 × 10^4^ cells per well). Lentiviral vectors were transfected into LUAD cells, where the MOI of PC-9 cells was 20 and the MOI of A549 cells was 10. The cells were collected 120 h after transfection for subsequent experiments. Viral vectors were designed by Shanghai Genechem Co., Ltd., including shTARS2-1 (5′-GGAGTGAGCAAGAGGAATA-3′), shTARS2-2 (5′-GATTTGACCTCCAGTATAA-3′) and scramble control(5′-TTCTCCGAACGTGTCACGT-3′) [23].

### 2.5. Western blotting

Fresh tissue and cell samples were lysed using RIPA buffer on ice. A 10% sodium dodecyl sulfate-polyacrylamide gel electrophoresis gel was used to separate the protein samples. The following antibodies were used in this study: TARS2 (15,067-1-AP, Proteintech, China); GAPDH (60,004-1-Ig, Proteintech, China); Phospho-RB (ser807/811) (9917, Cell Signaling Technology); Cyclin E1(20,808, Cell Signaling Technology); Cyclin E2 (4132, Cell Signaling Technology); CDK2 (18,048, Cell Signaling Technology); p21 Waf1/Cip1 (9932, Cell Signaling Technology); MT-ND6 (A17991, ABclonal, China); CYTB (55,090-1-AP, Proteintech, China); MT-CO1(62,101; Cell Signaling Technology); MT-CO3 (55,082-1-AP, Proteintech, China); MT-ATP8 (26,723-1-AP, Proteintech, China); TOM20 (11,802-1-AP, Proteintech, China); cleaved caspase-3 and cleaved caspase-8 (92,570, Cell Signaling Technology); cleaved caspase-9 (9930, Cell Signaling Technology) [24].

### 2.6. MTS assay

Scramble-, shTARS2-1, and shTARS2-2 transfected cells were inoculated into 96-well plates (1000 cells per well for A549 and 2000 cells per well for PC-9) with 200 μL of standard medium per well. Subsequently, 40 μL of MTS (G111A, Promega, Wisconsin, USA) was added to each well at 0 d, 1 d, 2 d, 3 d, 4 d, and 5 d and incubated in the dark for 120 min. Absorbance was measured at 490 nm [25].

### 2.7. Clone formation experiment

Scramble-, shTARS2-1-, and shTARS2-2-transfected cells were vaccinated in 6-well plates (200 cells/well). The transfected cells were cultured at 37°C for 14 days, and then fixed in paraformaldehyde for 30 min and stained with Giemsa (32,884; Sigma, Missouri, USA) for 10 min. Colonies were counted macroscopically [23].

### 2.8. EdU staining assay

Scramble-, shTARS2-1-, and shTARS2-2-transfected cells were seeded in 6-well plates (2 × 10^5^ cells/well) and stained with the EdU kit (Ribbio, China), and EdU-positive cells were counted under a fluorescent microscope (Nikon, Japan) [26].

### 2.9. mROS assay by flow cytometry

Scramble-, shTARS2-1-, and shTARS2-2-transfected cells were seeded in 6-well plates (2 × 10^5^ cells/well) and then cultured with MitoROS™ 580 (16,052, AAT Bioquest Inc., USA) for 30 min. mROS production was detected via flow cytometry (Ex/Em = 510/580) [27].

### 2.10. MMP detection

Scramble-, shTARS2-1-, and shTARS2-2-transfected cells were seeded in 6-well plates (2 × 10^5^ cells/well) and cultured with 1 mL JC-1 staining working solution (C2003S, Beyotime) for 20 min. The cells were later washed twice using PBS. Cells were photographed under an inverted fluorescence microscope (Nikon, Japan) [28,29].

### 2.11. Cell cycle and apoptosis

The cell cycle of samples was detected via flow cytometry using the Beyotime Cell Cycle Kit (C1052, Beyotime, Guangzhou), and sample apoptosis was detected via flow cytometry with Annexin V FITC/PI (556,547, BD, USA). The cells were starved for 8 h prior to cell cycle analysis [30].

### 2.12. Inhibition of mROS by melatonin

Next, we determined whether TARS2 knockdown induced apoptosis via mROS. shTARS2-1-transfected cells were cultured with 100 μM melatonin (M5250, Sigma, USA) for 2 h; mROS, MMP, and apoptosis were then detected using the above methods [31–33].

### 2.13. Nude mouse xenograft assay

Six-week-old, 18–22 g female BALB/c nude mice (SiPeiFu Biology Ltd., China) were randomly divided into two groups: scramble and shTARS2-1 groups of 6 mice each, and 5 × 10^6^ A549 cells were injected subcutaneously into the right axillary fossa of each nude mouse (the experiment was stopped when the tumor size affected the animals’ diet). Six weeks later, the mice were euthanized under anesthesia. Tumor volumes were measured weekly and evaluated using the following formula: length×width^2^/2. The experiments were approved by the Experimental Animal Ethics Committee of Kunming Medical University. All mice were housed in a temperature-controlled room (humidity, 50%±10%; temperature of 22 ± 2°C). The mice were allowed to drink and eat freely [11].

### 2.14. Statistical analysis

Statistical analysis of all data is expressed as the mean ± standard deviation (SD). A chi-squared test was used to analyze the correlation between TARS2 expression in LUAD tissues and clinicopathological parameters. OS rates were analyzed using the log-rank test (Kaplan-Meier plotter). A t-test was used to analyze statistical differences between the two groups. A parametric generalized linear model with random effects was used to analyze the MTS assay and tumor growth data. Statistical analyses were performed using SPSS v25.0 software. GraphPad Prism software (version 8.0) was used for to visualize the results. Statistical significance was set at p < 0.05 (* p < 0.05) [23].

## 3. Results

The purpose of this study was to explore the clinical significance and biological function of TARS2 in lung adenocarcinoma. We hypothesized that TARS2 may serve as a clinical diagnostic and prognostic marker and may be involved in tumor progression. To elucidate the correlation between TARS2 and clinically related pathological parameters, we analyzed the expression of TARS2 in lung adenocarcinoma and non-cancerous lung tissues in TCGA and CPTAC databases and tissue samples. To explore the role of TARS2 in LUAD, lentiviruses were used to knockdown TARS2 expression. Cell proliferation was detected using MTS, clone formation, and EdU staining assays. Flow cytometry was used to detect the cell cycle, mROS production, and apoptosis. ΔΨm was detected using a JC-1 fluorescent probe. Melatonin was used to reduce mROS production induced by TARS2 knockdown. Cell cycle, apoptosis-related pathways, and mtDNA-encoded protein expression were detected via Western blotting. Finally, the effect of TARS2 on tumor growth was examined using a xenotransplanted tumor model in nude mice.

### 3.1 TARS2 is upregulated in LUAD tissues

**Figure 1.**
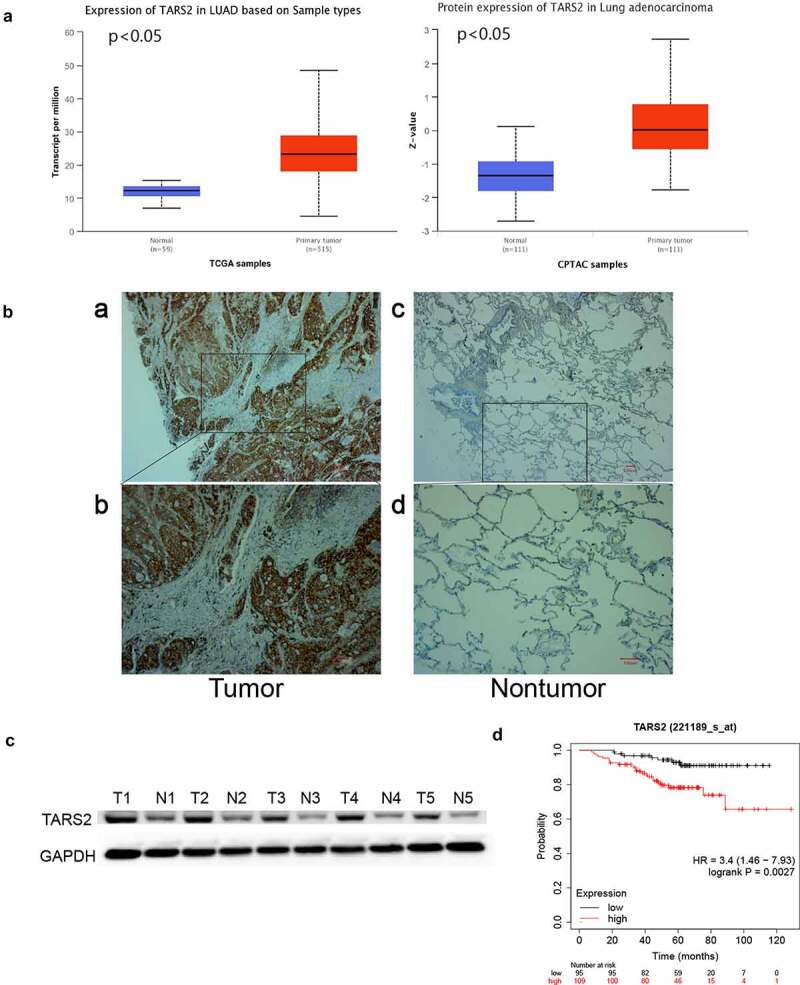
TARS2 is upregulated in LUAD tissues and associated with a poorer prognosis. (a) TARS2 mRNA levels were significantly increased in LUAD (TCGA dataset). CPTAC dataset analysis revealed that TARS2 protein levels were significantly increased in LUAD. (b) TARS2 expression in LUAD tissues, as detected by immunohistochemical staining to verify TARS2 expression in (a – b) LUAD tissue and paired (c – d) nontumor tissue; a – b, positive TARS2 staining in LUAD tissue; c – d, negative TARS2 staining in nontumor tissue. The scale bar represents 100 μm. (c) TARS2 expression in LUAD tissues was detected by Western blotting. (d) The Kaplan-Meier plotter database predicted the OS rates of LUAD patients with TARS2 expression.

By analyzing the dataset from TCGA and CPTAC (http://ualcan.path.uab.edu/analysis.html), we found that TARS2 mRNA and protein expression was upregulated in primary tumors (Figure 1(a)). TARS2 expression in 152 LUAD and 152 paired non-tumor tissues was detected using IHC (Figure 1(b)). TARS2 was located in the cytoplasm and was highly expressed in 152 lung adenocarcinoma tissues. (67.76% vs 30.92%, Table 1). Western blotting confirmed the high expression of TARS2 in five tumor tissues of lung adenocarcinoma patients (Figure 1(c)).

**Table 1.** Correlation between the clinicopathological characteristics and expression of TARS2 in LUAD

characteristics		n	TARS2 expression		*P* value
			Negative (n)	Positive (n)	
Tumor tissues		152	49(32.24%)	103(67.76%)	0.000*
Paired non-tumor tissues		152	105(69.08%)	47(30.92%)	
Age(y)					
	≤60	103	30(29.13%)	73(70.87%)	0.234
	>60	49	19(38.78%)	30(61.22%)	
Gender					
	Male	62	17(27.42%)	45(72.58%)	0.292
	Female	90	32(35.56%)	58(64.44%)	
Smoking history					
	Yes	38	9(23.68%)	29(76.32)	0.193
	No	114	40(35.09%)	74(64.91%)	
T stage					
	T_1_	102	30 (29.41%)	72(70.59%)	0.573
	T_2_	42	16(38.10%)	26(61.90%)	
	T_3_+ T_4_	8	3(37.5%)	5(62.5%)	
N classification					
	N_0_	96	35(36.46%)	61(63.54%)	0.145
	N_1+_N_2_	56	14(25.00%)	42(75.00%)	
AJCC stage					0.202
	I	88	32(36.36%)	56(63.64%)	
	II+III	64	17(26.56%)	47(73.44%)	

P < 0.05. TARS2, mitochondrial threonyl-tRNA synthetase 2.

### 3.2. Relation between TARS2 expression in LUAD tissues and clinical characteristics

By analyzing our data, we found that there was no statistically significant correlation between TARS2 expression and AJCC clinical-stage, sex, age, T stage, N classification or smoking history (Table 1). OS was poorer in LUAD patients with positive TARS2 expression than in those with negative TARS2 expression (patients with negative surgical margins were selected, http://kmplot.com/analysis/index p < 0.05, Figure 1(d)).

### 3.3. TARS2 knockdown inhibits proliferation of LUAD cells

**Figure 2.**
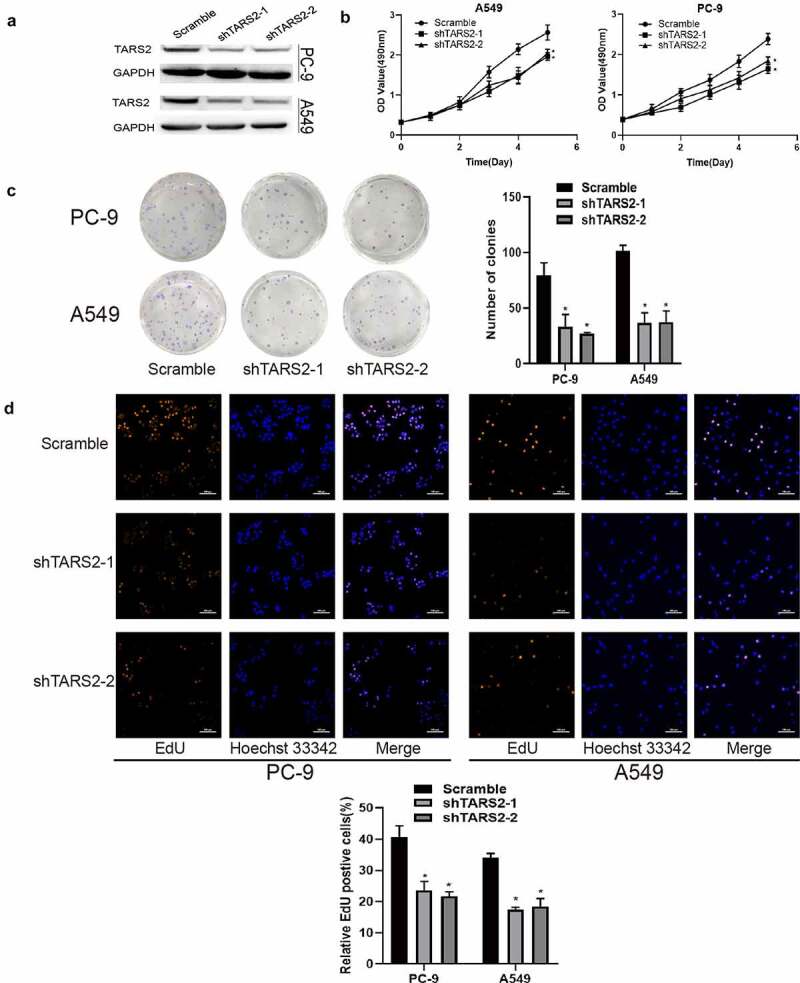
Knockdown of TARS2 inhibits the proliferation of LUAD cells. (a) TARS2 shRNA lentivirus decreased TARS2 expression. GAPDH is utilized as a reference protein. (b) TARS2 knockdown significantly inhibited the growth of LUAD cells, as evaluated by MTS. (c) In colony formation assays, TARS2 knockdown notably reduced the colony numbers of LUAD cells. (d) TARS2 knockdown notably reduces the ratio of EdU-positive LUAD cells (the scale bar represents 100 μm).

TARS2 shRNA was used to inhibit TARS2 expression in LUAD cells (Figure 2(a)). MTS analysis showed that the knockdown of TARS2 significantly inhibited the viability of LUAD cells (Figure 2(b)). A colony formation assay showed that TARS2 knockdown significantly reduced the number of colonies formed (Figure 2(c)). EdU analysis revealed that TARS2 knockdown caused a significant reduction in DNA synthesis in A549 and PC-9 cells (Figure 2(d)).

### 3.4. TARS2 gene regulates cell cycle through RB pathway

**Figure 3.**
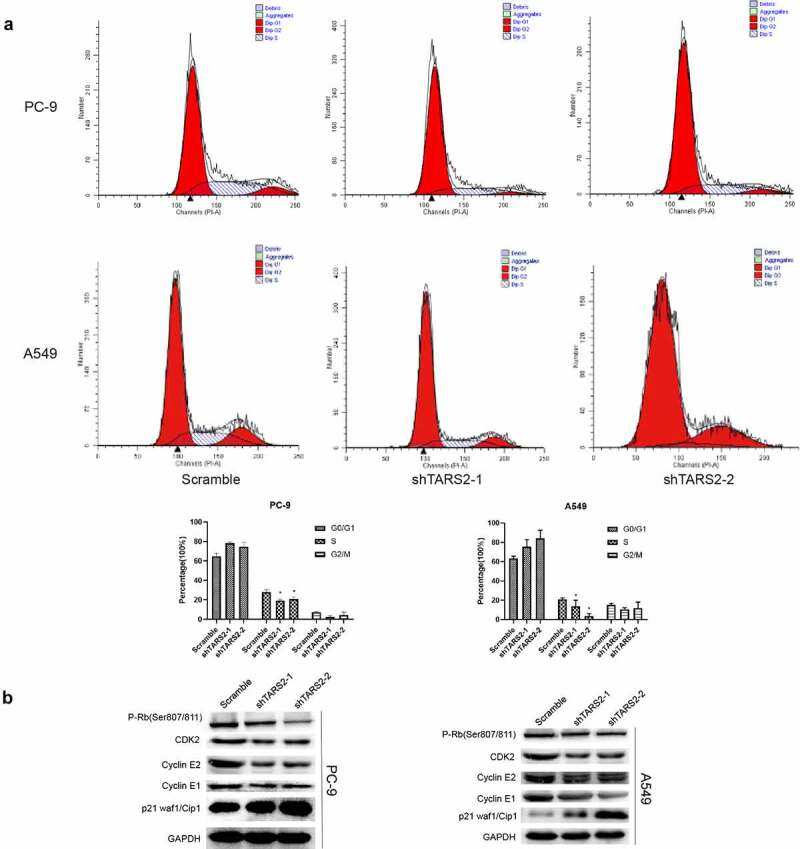
TARS2 knockdown inhibits cell cycle progression (a) Downregulation of TARS2 inhibits the transition from G1 phase to S phase, as evidenced by a flow cytometric assay. (b) The protein levels of p-RB (ser807/811), CDK2, cyclin E1 and cyclin E2 were decreased and that of p21 waf1/cip1 was increased in TARS2-shRNA transfected LUAD cells. GAPDH is utilized as a reference protein.

Flow cytometry demonstrated that knockdown of TARS2 reduced the percentage of cells in the S phase in LUAD cells (Figure 3(a)). Cyclin-dependent kinases (CDKs) combines with a specific cyclin to control cell cycle progression [34]. Cyclin E combines with CDK2 to form a complex and promote retinoblastoma protein phosphorylation [35,36]. Phosphorylated RB (p-RB) releases E2F, which activates the transcription of S-phase genes and promotes G1/S transition [37]. In contrast, p21 Waf1/Cip1, a cell cycle protein-dependent kinase (CDK) inhibitor, prevents G1/S transition by inhibiting cyclin E [38]. To elucidate the mechanism by which knockdown of the TARS2 gene can regulate the cell cycle and inhibit cell proliferation, we detected G1/S phase checkpoint proteins via Western blotting and found that p-RB, cyclin E1, cyclin E2, and CDK2 expression decreased and p21 expression increased after knockdown of TARS2 (Figure 3(b)). Our results suggest that TARS2 regulates the cell cycle via the RB pathway.

### 3.5. TARS2 knockdown induces mitochondrial reactive oxygen species production by reducing mitochondrial-DNA expression

**Figure 4.**
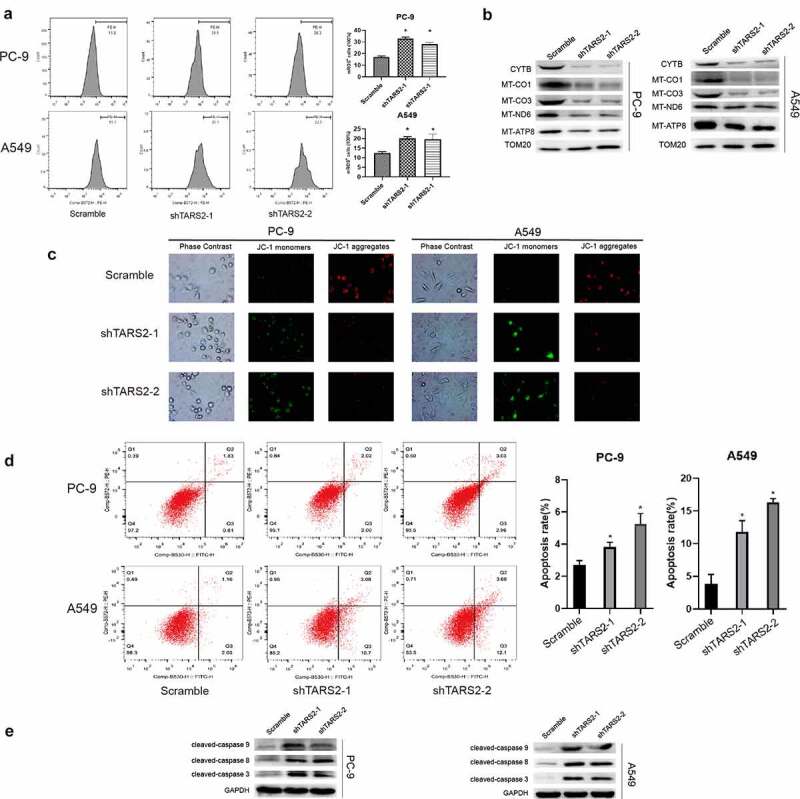
TARS2 knockdown and increases mROS-induced apoptosis of LUAD cells. (a) Downregulation of TARS2 increased production of mROS in PC-9 and A549. (b) The expression of MT-ND6, CYTB, MT-CO1, MT-CO3 and MT-ATP8 were decreased in TARS2-shRNA transfected LUAD cells. TOM20 is utilized as a reference protein. (*P < 0.05). (c) Downregulation of TARS2 decreases mitochondrial membrane potential in A549 and PC-9. (d) Downregulation of TARS2 increases PC-9 and A549 cells apoptosis. (e) The protein levels of cleaved caspase-8, −9 and −3 were decreased in TARS2-shRNA transfected LUAD cells. GAPDH is utilized as a reference protein. (*P < 0.05).

We found an increase in mitochondrial reactive oxygen species (mROS) production after knockdown of TARS2 by flow cytometry analysis (*p < 0.05, Figure 4(a)). ROS are mainly produced in the mitochondria and mutation or loss of mitochondrial DNA can lead to overproduction of ROS. TARS2 is located in mitochondria and participates in protein translation. We speculated that TARS2 knockdown increases the production of mitochondrial ROS, possibly due to the downregulation of mitochondria-DNA (mtDNA). The results of Western blotting confirmed this conjecture; knockdown of TARS2 decreased the expression of MT-ND6, CYTB, MT-CO1, MT-CO3, and MT-ATP8 (Figure 4(b)).

### 3.6. TARS2 knockdown decreases mitochondrial membrane potential (MMP, ΔΨm) and induces apoptosis of LUAD cells

MMP is an important indicator of mitochondrial function [39]. We assessed the effect of TARS2 knockdown on MMP using JC-1 staining. Our results showed that the knockdown of TARS2 increased the conversion of JC-1 aggregates to monomers (Figure 4(c)). These findings revealed that TARS2 knockdown decreased the MMP in LUAD cells. Decreased MMP is a signal of early apoptosis [40]. ROS can induce apoptosis in various cell types. We found an increase in apoptosis after TARS2 knockdown by flow cytometry analysis (*p < 0.05, Figure 4(d)). The expression of apoptotic proteins cleaved-caspase-8, −9, and −3 increased after knockdown of TARS2 (Figure 4(e)). These results suggest that the knockdown of TARS2 promotes ROS-induced apoptosis in LUAD cells.

### 3.7. Inhibition of mROS production reduces apoptosis induced by TARS2 knockdown

**Figure 5.**
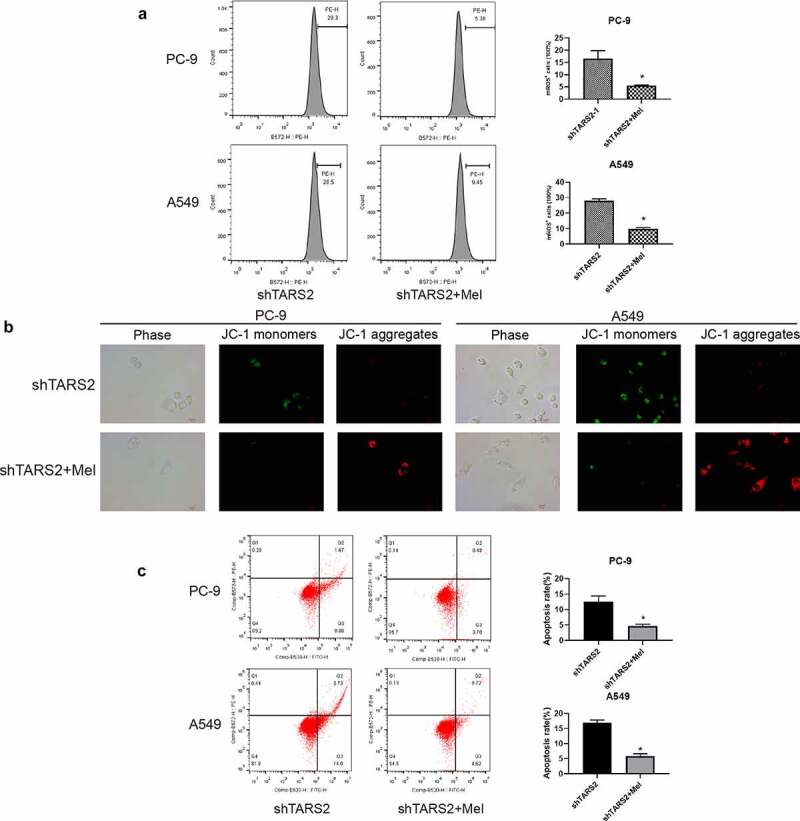
Inhibition of mROS production by melatonin protects MMP and reduces apoptosis. (a) Melatonin decreases mROS production induced by TARS2 knockdown. (b) Melatonin protects MMP induced by TARS2 knockdown. (c) Melatonin protects LUAD from apoptosis induced by TARS2 knockdown. (*P < 0.05).

We detected mROS production in shTARS2-1-transfected cells co-cultured with melatonin and found that melatonin could significantly inhibit the mROS production caused by TARS2 knockdown (Figure 5(a)). We assessed the impact of mROS production on MMP through JC-1 staining and found that inhibition of mROS production could protect MMP in shTARS2-1-transfected A549 and PC-9 cells (Figure 5(b)). The flow cytometry results demonstrate that inhibiting mROS production significantly reduces apoptosis induced by TARS2 knockdown (Figure 5(c)). These results show that mROS are the key to apoptosis induced by TARS2 knockdown.

### 3.8. TARS2 knockdown inhibits LUAD growth in vivo

**Figure 6.**
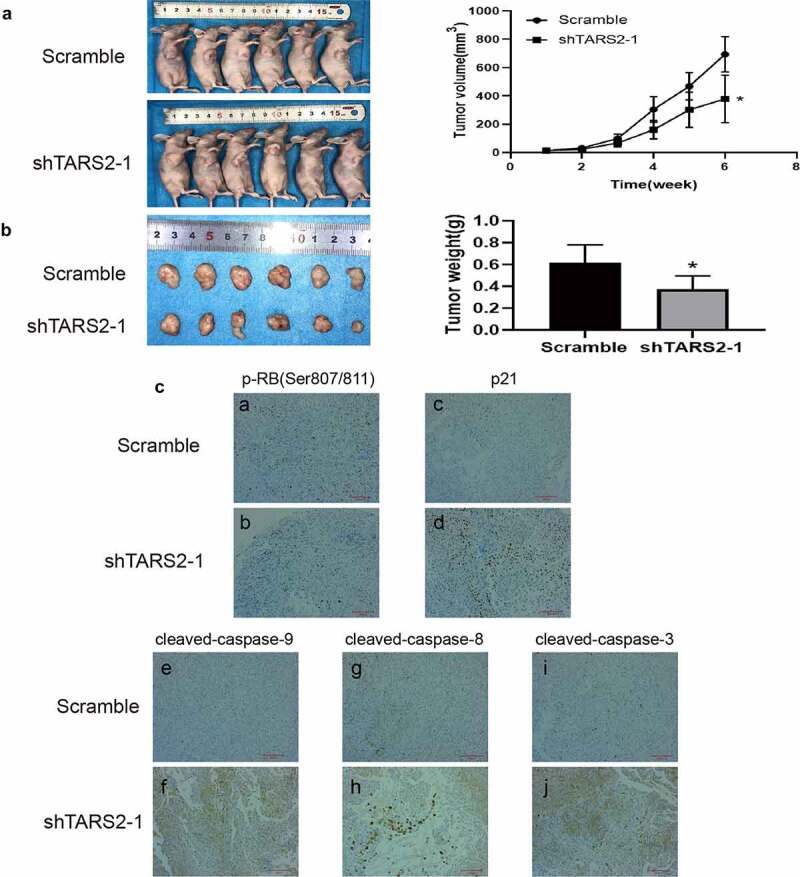
Knockdown of TARS2 inhibits LUAD cells growth in vivo. (a) Tumor growth curves of mice inoculated with A549 cells infected with TARS2 lentivirus were measured weekly with vernier calipers. (b) Tumor weights at 6 weeks post injection. (c)Protein expression in tumor tissue sections detected by immunohistochemical staining. a – b, p-RB(Ser807/811) expression was decreased in shTARS2-1 group compared with SCRAMBLE group; c – d p21 expression was increased in shTARS2-1 group compared with SCRAMBLE group; e-f, cleaved-caspase 9 expression was increased in shTARS2-1 group compare with SCRAMBLE group; g-h, cleaved-caspase 8 expression was increased in shTARS2-1 group compare with SCRAMBLE group; i-j, cleaved-caspase 3 expression was increased in shTARS2-1 group compare with SCRAMBLE group. The scale bar represents 200 μm.

The results of the mouse subcutaneous tumor model indicated that the tumor growth rate of A549 cells transfected with shTARS2-1 was significantly inhibited compared with that of cells transfected with scramble shRNA. Tumor size and weight were lower in the shTARS2-1 group than in the scrambled control group (Figure 6(a,b)). The results of immunohistochemical staining showed that TARS2 knockdown decreases p-RB (ser807/811) expression and increases p21, cleaved-caspase-3, −8 and −9 expression (Figure 6(c)). These results suggest that the knockdown of TARS2 suppresses A549 cell growth by inhibiting proliferation and inducing apoptosis.

## 4. Discussion

TARS and TARS2 are both members of the aaRS family and encode cytoplasmic and mitochondrial threonyl-tRNA synthetases, respectively. AaRSs take part in tumor proliferation, invasion, metastasis, angiogenesis, and prognosis [12,41–44]. TARS is associated with tumor angiogenic markers and metastasis and affects the prognosis of patients with tumors. However, no tumor-related studies of TARS2 have been reported.

Our study showed that TARS2 was highly expressed in LUAD and was related to poor OS in patients. These results suggest that the upregulation of TARS2 is a potential prognostic factor for LUAD.

Previous reports have shown that TARS2 is required for threonine-sensitive mTORC1 activation in HEK293T cells [45]. mTORC1 is a central regulator of cell proliferation [46]. We suspect that TARS2 may play a role in cell proliferation. To elucidate the biological function of TARS2 in LUAD, shRNA lentiviruses were used to decrease TARS2 expression. Functional assays revealed that the knockdown of TARS2 in LUAD cells inhibited cell proliferation.

Excessive cell proliferation is a crucial biological feature of tumors and the level of cell proliferation is closely related to cell cycle progression [47]. Our results showed that the knockdown of TARS2 in LUAD decreased the expression of p-RB, cyclin E1, cyclin E2, and CDK2, while the expression of p21 increased. Flow cytometry and EdU assays revealed a decrease in the percentage of cells in the S phase. Thus, we can infer that TARS2 knockdown inhibits cell proliferation through the RB pathway in LUAD cells.

Mitochondria have long been thought to provide energy to the body under aerobic conditions. More recently, mitochondria have been shown to play a key role in invasion, apoptosis, cell proliferation, and anti-tumor therapy in tumors [48]. mtDNA encodes many critical proteins involved in respiratory complexes [49]. TARS2 is located in the mitochondria and participates in protein translation, and mutations in TARS2 cause mitochondrial encephalomyopathies [18]. mtDNA mutation or loss is a vital characteristic of mitochondrial encephalomyopathies [20]. These studies indicate that TARS2 may participate in mitochondrial function by regulating mtDNA translation. Western blotting results indicated that knockdown of TARS2 in LUAD decreased the expression of NADH dehydrogenase subunit 6 (ND6), mitochondrial cytochrome b (CYTB), mitochondrially encoded cytochrome c oxidase 1 (MT-CO1), mitochondrially encoded cytochrome c oxidase 3 (MT-CO3), and mitochondrially encoded ATP synthase 8 (MT-ATP8). ND6 is a subunit of complex I, CYTB is a subunit of complex III, MT-CO1 and MT-CO3 are subunits of complex IV, and MT-ATP8 is a subunit of complex V [50–53].

The mitochondrial respiratory enzyme complex regulates the mitochondrial electron transport chain. A previous study showed that reactive oxygen species are produced when the respiratory enzyme complex is dysfunctional [54–56]. Ishikawa et al. showed that the G13997A mutation in ND6 induces an overproduction of ROS in tumor cells [57]. Gonzalo et al. showed that the ND6 T14487C mutation causes overproduction of ROS [58]. Sally A. Madsen–Bouterse et al. and Julia Matzenbacher et al. showed that decreased CYTB transcription promotes ROS production in mice [59,60]. Heike Weiss et al. demonstrated that an FVB mutation in ATP8 induces ROS generation [61]. Our flow cytometry results demonstrate that TARS2 knockdown increases mROS production. mtDNA plays an important role in mitochondrial function. MMP is an important indicator of mitochondrial function. Many studies have shown that mROS induces mitochondrial membrane potential depolarization [31]. We assessed the effect of TARS2 knockdown on MMP using JC-1 staining. Our results show that TARS2 knockdown decreases MMP in PC-9 and A549 cells.

Decreased MMP induces apoptosis by releasing cytochrome c into the cytoplasm, which signals early apoptosis. Cancer is a condition whereby apoptosis is too low, resulting in malignant cells that do not die [62–64]. Caspases play a crucial role as important execution factors in apoptosis [63,65]. Caspase-8 and caspase-9 are both members of initiator caspases and participate in extracellular and intracellular apoptosis signals, respectively. ROS induces apoptosis by activating caspase-8 and caspase-9, and this activation may not be direct [66–70]. Caspase-3 is an effector caspase that can be activated by caspase-8 and −9 to induce apoptosis [71]. Flow cytometry results showed that the knockdown of TARS2 induced apoptosis in LUAD cells. The results of Western blotting showed that knockdown of TARS2 in A549 and PC elevated the expression of pro-apoptotic proteins cleaved caspase-9, −8, and −3.

Melatonin is an antioxidant produced in the pineal gland and can protect cells from oxidative damage [32]. To study whether mROS participate in TARS2 knockdown-induced apoptosis, melatonin was used to reduce mROS generation. Our results showed that melatonin reduced oxidative stress induced by TARS2 knockdown (Figure 5(a)). JC-1 staining showed that melatonin protected mmp in shTARS2-1-transfected A549 and PC-9 cells (Figure 5(b)). The results of flow cytometry showed that inhibiting mROS generation significantly reduced apoptosis induced by TARS2 knockdown. These results confirm that TARS2 modulates ROS-induced apoptosis through mtDNA. Animal experiments have shown that the knockdown of TARS2 inhibits the growth of transplanted tumors.

## 5. Conclusions

In summary, TARS2 expression significantly increased in patients with LUAD and was associated with a poorer prognosis. Knockdown of TARS2 inhibits cell proliferation via the RB pathway and promotes ROS-induced apoptosis. Thus, TARS2 plays a carcinogenic role and may be a therapeutic target for LUAD.

## Research highlights

- TARS2 is highly expressed in LUAD and associated with poorer patient prognosis.
- Knockdown of TARS2 inhibits cell proliferation through the RB pathway and promotes ROS-induced apoptosis.
- Knockdown of TARS2 decreases the expression of mtDNA and increases the production of mROS in LUAD.
